# Refining the Multivariable Predictive‐Prognostic PREDICTR‐OPC Model for Survival in Surgical Escalation for Oropharyngeal Squamous Cell Carcinoma

**DOI:** 10.1002/lary.70611

**Published:** 2026-05-12

**Authors:** Lauren L. Zhang, Caroline Kristunas, C. Max Robinson, Jill M. Brooks, Alice J. Sitch, Stuart C. Winter, Justin Weir, Paul Matthews, Terry M. Jones, Keith Hunter, Pawel Golusinski, Ketan A. Shah, Selvam Thavaraj, Catharine M. West, Syed Haider, Edward Odell, Paul Nankivell, Sandra V. von Zeidler, Hisham Mehanna

**Affiliations:** ^1^ Columbia University Vagelos College of Physicians and Surgeons New York New York USA; ^2^ Department of Cancer and Genomic Sciences, University of Birmingham Institute of Head and Neck Studies and Education (InHANSE) Birmingham UK; ^3^ Department of Cellular Pathology Newcastle Upon Tyne Hospitals NHS Foundation Trust Newcastle Upon Tyne UK; ^4^ Institute of Applied Health Research, University of Birmingham Birmingham UK; ^5^ NIHR Birmingham Biomedical Research Centre, University Hospitals Birmingham NHS Foundation Trust Birmingham UK; ^6^ Nuffield Department of Surgical Sciences University of Oxford Oxford UK; ^7^ Department Cellular Pathology Royal Marsden Hospital London UK; ^8^ Department of Histopathology University Hospitals Coventry and Warwickshire Coventry UK; ^9^ Liverpool Head and Neck Centre, University of Liverpool Liverpool UK; ^10^ Department of Otolaryngology and Maxillofacial Surgery University of Zielona Góra Zielona Góra Poland; ^11^ Department of Maxillofacial Surgery Poznan University of Medical Sciences Poznan Poland; ^12^ Department of Cellular Pathology John Radcliffe Hospital Oxford UK; ^13^ Department of Oral & Maxillofacial Clinical Sciences University of Malaya Kuala Lumpur Malaysia; ^14^ Translational Radiobiology Group, Division of Cancer Sciences University of Manchester, Manchester Cancer Research Centre, Christie NHS Foundation Trust Manchester UK; ^15^ Breast Cancer Now Toby Robins Breast Cancer Research Centre, the Institute of Cancer Research London UK; ^16^ King's College London; Head and Neck Pathology, Guy's and St Thomas' Hospitals NHS Foundation Trust London UK; ^17^ Department of Otolaryngology Queen Elizabeth Hospital Birmingham UK; ^18^ Department of Pathology Federal University of Espirito Santo Vitoria Brazil

**Keywords:** human papillomavirus, oropharyngeal squamous cell carcinoma, prognostic model, surgical escalation

## Abstract

**Objectives:**

The PREDICTR‐OPC model is the only prognostic classifier for oropharyngeal squamous cell carcinoma (OPSCC) also predictive of surgical outcomes. Of the four biomarkers included, survivin contributes minimally and presents practical limitations. This study aimed to refine and simplify the model by removing survivin, then re‐assess its prognostic predictive performance compared to the original.

**Methods:**

This retrospective cohort study analyzed a multi‐center training cohort (*n* = 600) and an external validation cohort (*n* = 385) of OPSCC patients. Tumor biopsies were stained for p16, high‐risk human papillomavirus (HR‐HPV) DNA, tumor‐infiltrating lymphocytes (TILs), and survivin and independently scored by at least three certified pathologists. Cox proportional hazards models assessed overall survival (OS), comparing three‐biomarker (p16, HR‐HPV, TILs) and four‐biomarker models. Hazard ratios (HRs) for OS were estimated in the validation cohort, adjusting for covariates. Discrimination, calibration, and decision curve analysis (DCA) evaluated performance and clinical utility.

**Results:**

Among 985 patients (median age: 57 years), median OS = 8.8 years (95% CI: 6.9–10.5). The three‐biomarker model yielded HR = 4.10 (95% CI: 2.41–6.98, *p* < 0.001) for high‐ vs. low‐risk groups in the validation cohort, comparable to the four‐biomarker model (HR = 4.24, *p* < 0.001). Surgery was associated with improved OS in high‐risk (HR = 0.45, *p* = 0.001) but not low‐risk (HR = 0.83, *p* = 0.72) patients, consistent with the original model.

The models performed similarly across all metrics (e.g., Concordance Index: 0.71 vs. 0.72; Brier Score: 0.22 for both) as was model fit (Likelihood Ratio Test: *p* = 0.066). DCA revealed comparable clinical benefit.

**Conclusion:**

Removing survivin preserves PREDICTR‐OPC's predictive performance, offering a more cost‐effective, easier‐to‐implement tool for OPSCC treatment recommendations.

**Level of Evidence:**

3

## Graphical abstract

The PREDICTR‐OPC model is the only prognostic classifier for oropharyngeal squamous cell carcinoma (OPSCC) that also predicts surgical outcomes. This study demonstrates that removing survivin from the model preserves its prognostic and predictive performance, with comparable discrimination, calibration, and clinical utility across training and validation cohorts. The simplified three‐biomarker model offers a more cost‐effective and practical tool for guiding surgical escalation in OPSCC treatment.

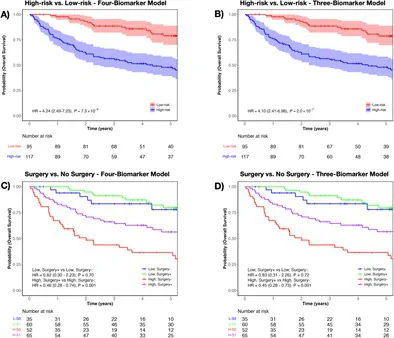

## 1 Introduction

In recent years, the incidence of oropharyngeal squamous cell carcinoma (OPSCC) has increased in the US, UK, and Western Europe, largely attributed to Human papillomavirus (HPV) [1, 2, 3]. HPV‐associated OPSCC demonstrates significantly higher 5‐year overall survival (OS) (80% vs. 40%–50% for HPV‐negative disease) [4, 5, 6]. Conventional treatment for OPSCC is by chemoradiotherapy (CRT), but transoral surgery with adjuvant treatment has recently become widespread [7, 8]. Treatment decisions are currently guided by clinical judgment and patient preferences. While models for risk stratification exist, these are prognostic and are mostly not predictive for treatment selection [6].

To address this, Mehanna et al. developed the PREDICTR‐OPC model, which stratifies OPSCC patients into risk categories based on OS probabilities and is predictive for treatment selection in high‐risk patients [9]. The original classifier incorporated four objective biomarkers: p16 and survivin immunohistochemistry (IHC); high‐risk HPV (HR‐HPV) in situ hybridization (ISH); and tumor‐infiltrating lymphocytes (TILs), quantified on hematoxylin and eosin (H&E) slides [10].

HR‐HPV detection confirms viral etiology [11]. p16 overexpression reflects viral oncoprotein activity and is independently associated with improved survival [12, 13]. TILs reflect the host immune response, with higher levels indicating enhanced anti‐tumor immunity, associated with improved survival [14]. Survivin, an inhibitor of apoptosis, is linked to tumor aggressiveness, treatment resistance, and poorer prognosis [10, 15, 16].

PREDICTR‐OPC demonstrated robust prognostic performance, with p16, HR‐HPV, and TILs being the main contributors. By contrast, survivin was found to provide only a small contribution to the model and was the only biomarker for which automated staining was not possible. Also, survivin scoring was confounded by moderate interobserver variability, negatively impacting reproducibility and clinical scalability.

Thus, this study aims to optimize the PREDICTR‐OPC model by removing survivin and comparing prognostic risk stratification, predictive ability for treatment escalation, and clinical utility of the three‐biomarker model (p16, HR‐HPV, TILs) with the original four‐biomarker classifier.

## 2 Methods

### 2.1 Study Design and Data

This retrospective cohort study used two previously described datasets: a model development (training) cohort of 600 OPSCC patients treated with curative intent across ten centers in the United Kingdom and Poland (1999–2012), and an independent external validation cohort of 385 patients from the HPV UK Prevalence study (2002–2011) [9, 17]. Clinicians blinded to the biomarker analysis collected baseline, treatment, and outcome data from health records. Tissue microarrays were constructed from formalin‐fixed, paraffin‐embedded diagnostic tumor biopsies which were then stained and scored independently by at least three pathologists. p16 IHC and HR‐HPV ISH were categorized as positive or negative; TILs as high, moderate, or low; and survivin IHC quantified as a continuous H‐score (0–300), divided by 30 and *z*‐transformed. The primary outcome was OS, defined as time from diagnosis to death or censoring at last follow‐up. Full staining, scoring, and classification protocols were reported previously [9].

### 2.2 Statistical Analysis

To compare risk stratification and predictive ability, we estimated hazard ratios (HRs) between risk groups and treatment strategies using Cox proportional hazards models. OS probabilities were estimated and visualized using Kaplan–Meier curves. Discrimination, calibration, overall performance, and clinical utility metrics were assessed, following STRengthening Analytical Thinking for Observational Studies (STRATOS) initiative recommendations [18]. To assess the effect of missing data, the three‐biomarker model was retrained on imputed datasets, and compared the complete‐case validation results. All analyses were conducted in R (version 4.4.3).

#### 2.2.1 Model Training and Evaluation

Two Cox proportional hazards models were constructed: the four‐biomarker model (p16, HR‐HPV, survivin, and TILs) and the refined three‐biomarker model excluding survivin. Survival was compared with the log‐rank test. Each model was trained on the multi‐center training cohort (*n* = 600), generated risk scores from linear predictors, and classified participants as high‐risk if their risk score was greater than or equal to the training cohort median (see Supporting Information).

#### 2.2.2 Survival Analysis of Risk Stratification and Treatment Strategy

The three‐ and four‐biomarker models fitted on the training cohort were used to estimate HRs for OS in high‐ vs. low‐risk groups in the independent external validation cohort (*n* = 385), adjusted for age, year of diagnosis, T‐category, N‐category, smoking status, surgery, radiotherapy, and chemotherapy. Participants with unknown or incomplete treatment records were included in overall survival analyses but were not eligible for treatment‐specific comparison analyses. To compare OS for surgical vs. non‐surgical treatment within risk groups, propensity score matching was performed to adjust for treatment bias due to baseline differences in T‐category, N‐category, smoking status, and age at diagnosis (see Supporting Information). Weighted Cox models were used to estimate marginal HRs for surgery vs. no surgery within risk groups. Kaplan–Meier curves with log‐rank tests compared 5‐year OS between treatments.

To address potential heterogeneity within the surgical cohort, we performed a sensitivity analysis restricted to patients who had received CRT only to compare with those who received trimodality therapy (surgery + CRT). Cox proportional hazards models were used to compare OS within high‐ and low‐risk groups as defined by the three‐ and four‐biomarker models.

### 2.3 Model Comparison Metrics

#### 2.3.1 Discrimination

Model discrimination, the ability to distinguish between high‐ and low‐risk patients, was assessed using Harrell's Concordance Index (C‐index); Uno's C‐index, adjusted for censoring; and the area under the receiver operating characteristic curve (AUROC) at 3 and 5 years. The C‐index measures overall predictive accuracy, while AUROC assesses discrimination at specific time points. Higher values indicate better discrimination (1 being perfect, 0.5 representing no better than chance) [19].

#### 2.3.2 Calibration

Calibration, the agreement between predicted survival probabilities and observed outcomes, was evaluated at the mean, weak, and moderate levels. Mean calibration, reflecting systematic under‐ or overprediction, was found with the observed‐to‐expected (O/E) ratio for 5‐year mortality; values closer to 1 indicate better calibration. Weak calibration, evaluating alignment across risk scores, was assessed with the calibration slope from a regression model with risk score as the only covariate and 5‐year administrative censoring. Moderate calibration evaluates agreement within subgroups of similar predicted risk, and was examined using smooth calibration curves [18]. For comparison, the Integrated Calibration Index (ICI) (mean absolute difference between observed and predicted probabilities) and median (E50) and 90th percentile (E90) of the absolute difference were calculated.

#### 2.3.3 Model Fit and Accuracy

Sensitivity, specificity, positive predictive value (PPV), and negative predictive value (NPV) were calculated for both models in the validation cohort. Model fit was compared using the likelihood ratio test; *p* < 0.05 indicates that the additional predictor (survivin) confers significant predictive value [20, 21]. Overall accuracy was compared using the Brier score (mean squared error between predicted and actual outcomes; lower scores indicate better performance) [22, 23], and the scaled Brier score or Index of Prediction Accuracy (IPA) (higher scores indicate better performance) [24]. Details are provided in the Supporting Information.

### 2.4 Decision Curve Analysis

While metrics such as calibration and discrimination assess model performance, they do not reflect clinical utility. To address this, we performed decision curve analysis (DCA), which quantifies the net benefit of using each model across a range of threshold probabilities (*p*_t_) [25, 26].

Net benefit incorporates the relative value of true‐ versus false‐positive classifications, reflecting the trade‐off between treatment benefit and harm. Here, *p*_t_ represents the predicted 5‐year mortality risk above which a clinician would consider surgical escalation plus adjuvant therapy; higher *p*_t_ values correspond to more conservative strategies, for example, if the side effects of surgery would be less tolerable [27, 28]. Conversely, lower *p*_t_ values imply a lower threshold for intervention and greater tolerance for overtreatment.

Decision curves plotting net benefit against *p*_t_ for both models were compared with default strategies of always escalating to surgery (treat‐all) and never escalating (no‐treatment). Ranges of *p*_t_ where net benefit was significantly higher than the defaults (*p* < 0.05) were identified by bootstrapping. See Supporting Information for details.

## 3 Results

### 3.1 Participant Characteristics

There were 985 participants in total, with 600 (60.9%) in the training cohort and 385 (39.1%) in the validation cohort (Table 1). The overall median age at diagnosis was 57 years, with a range of 19–91 years. 385 (39.1%) participants had T3–T4 disease and 373 (37.9%) had N2b–N3 disease; all staging followed the 7th edition of the American Joint Committee on Cancer (AJCC) and the Union for International Cancer Control (UICC) staging system [9]. In total, 439 (44.6%) participants underwent surgery; of these, 240 (24.4%) received adjuvant radiotherapy, 144 (14.6%) received adjuvant CRT, and 14 (1.4%) received unknown adjuvant treatment. 330 (33.5%) participants received primary CRT with platinum, 83 (8.4%) received radiotherapy alone, 10 (1.0%) received chemotherapy alone, and 11 (1.1%) received neither; 33 (3.4%) were unknown. The median OS was 8.8 years (95% CI: 6.9–10.5 years) and the 5‐year survival rate was 62.2%, with median follow‐up of 5.03 years (range: 4.73–5.21 years).

**TABLE 1:** Characteristics of the training and validation cohorts.

	Training cohort number (%)	Validation cohort number (%)	*p*_adj_ ^a^
	600 (60.9)	385 (39.1)	
Mean age, years (SD)	57.5 (10.5)	58.2 (10.5)	0.685
Gender			< 0.001
Male	434 (72.3)	241 (62.6)	
Female	161 (26.8)	85 (22.1)	
N/A	4 (0.7)	57 (14.8)	
Tumor category^b^			< 0.001
T1–T2	322 (53.7)	179 (46.5)	
T3–T4	249 (41.5)	136 (35.3)	
N/A	29 (4.8)	70 (18.2)	
Nodal category^b^			< 0.001
N0–N2a	328 (54.7)	184 (47.8)	
N2b–N3	248 (41.3)	125 (32.5)	
N/A	24 (4.0)	76 (19.7)	
Smoking history			0.0199
Current	200 (33.3)	137 (35.5)	
Past	163 (27.1)	92 (23.8)	
Never	142 (23.6)	69 (17.9)	
N/A	95 (15.8)	87 (22.5)	
Treatment			
Radiotherapy	548 (91.3)	280 (72.7)	< 0.001
Chemotherapy	358 (59.7)	133 (34.5)	< 0.001
Surgery	264 (44.0)	175 (45.5)	< 0.001
Recurrence			0.630
Censored	352 (58.7)	237 (61.5)	
Events	142 (23.7)	79 (20.5)	
N/A	106 (17.6)	69 (18.0)	
Overall survival			< 0.001
Censored	373 (62.2)	168 (43.6)	
Events	193 (32.2)	144 (37.4)	
N/A	34 (5.6)	73 (19.0)	
Survivin, mean H‐score (SD)	62.9 (37.6)	71.9 (36.9)	0.00117
p16			0.125
Negative	161 (26.8)	123 (31.9)	
Positive	439 (73.2)	262 (68.1)	
HR‐HPV DNA			0.0508
Negative	368 (61.3)	245 (63.6)	
Positive	160 (26.7)	113 (29.4)	
N/A	72 (12.0)	27 (7.0)	
TILs			< 0.001
1	89 (14.8)	67 (17.4)	
2	192 (32.0)	177 (45.9)	
3	119 (19.8)	94 (24.4)	
N/A	200 (33.3)	47 (12.2)	

^a^ *p*‐values were adjusted for multiple comparisons with the Benjamini‐Hochberg method.

^b^ TNM staging was performed according to guidelines in the AJCC/UICC TNM 7th edition manual.

### 3.2 Model and Risk Stratification

In the training cohort (*n* = 259), the three‐biomarker model yielded an HR for the primary outcome, OS, of 7.89 (95% CI: 4.03–15.44, *p* < 0.001) for high‐ vs. low‐risk groups, similar to that of the four‐biomarker model (HR = 8.20, 95% CI: 4.19–16.05, *p* < 0.001) (Figure S1). Model coefficients and survival curves are provided in Table S1 and Figure S2. Performance of the three‐biomarker model trained on imputed datasets was comparable to that of the complete‐case validation set (*n* = 212) used for final analysis (Table S2).

**FIGURE 1:**
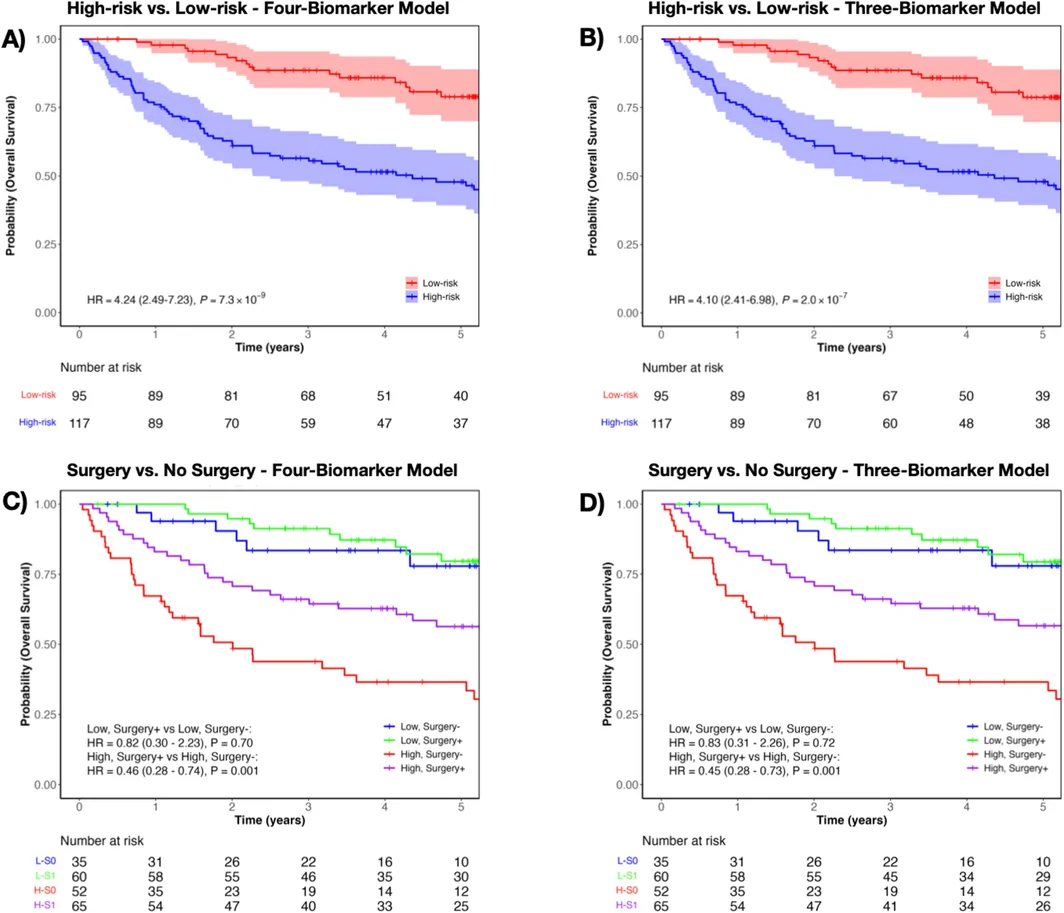
Kaplan–Meier survival curves comparing OS of the high‐ and low‐risk groups from the four‐biomarker (A) and three‐biomarker model (B) in the validation cohort; Kaplan–Meier survival curves comparing OS for treatment strategies (surgery vs. no surgery) within risk groups classified by the four‐biomarker model (C) and three‐biomarker model (D) in the validation cohort. [Color figure can be viewed in the online issue, which is available at www.laryngoscope.com]

In the validation cohort (*n* = 212), HRs for high‐ vs. low‐risk groups were similar between models: 4.10 (95% CI: 2.41–6.98, *p* < 0.001) for the three‐biomarker and 4.24 (95% CI: 2.49–7.23, *p* < 0.001) for the four‐biomarker model (Table 2). Kaplan–Meier curves are displayed in Figure 1. For the high‐risk group, the HR from the three‐biomarker model for those who underwent surgery and adjuvant treatment compared to CRT alone was 0.45 (95% CI: 0.28–0.73, *p* = 0.001). There was no significant difference in the low‐risk group (HR = 0.83, 95% CI: 0.31–2.26, *p* = 0.72), consistent with that of the four‐biomarker model (HRs of 0.46, 95% CI: 0.28–0.74, *p* = 0.001 and 0.82, 95% CI: 0.30–2.23, *p* = 0.70 respectively) (Figure 1). Log‐rank *p* < 0.001 in all comparisons indicate significant differences in survival distribution between risk groups for both models.

**TABLE 2:** Performance measures, survival metrics, discrimination and calibration in the four‐ and three‐biomarker models.

Measure	Four‐biomarker model	Three‐biomarker model
Overall model performance		
Overall sensitivity	0.80 (0.72–0.89)	0.80 (0.72–0.89)
Overall specificity	0.61 (0.53–0.70)	0.61 (0.53–0.70)
Overall PPV	0.58 (0.50–0.67)	0.58 (0.50–0.67)
Overall NPV	0.82 (0.74–0.90)	0.82 (0.74–0.90)
Five‐year survival (%)		
High risk	47.8	48.0
Low risk	78.9	78.7
Overall survival, HR (95% CI)		
Low risk	Reference	Reference
High risk	4.24 (2.49–7.23)*	4.10 (2.41–6.98)*
Overall survival (low risk), HR (95% CI)		
CRT only	Reference	Reference
Surgery + adjuvant therapy	0.82 (0.30–2.23)	0.78 (0.35–1.72)
Overall survival (high risk), HR (95% CI)		
CRT only	Reference	Reference
Surgery + adjuvant therapy	0.46 (0.28–0.74)*	0.42 (0.25–0.70)*
Discrimination, 5 years		
Uno's C‐index, validation set	0.72 (0.66–0.78)	0.71 (0.65–0.76)
Harrell's C‐index, validation set	0.73 (0.68–0.79)	0.72 (0.67–0.78)
Uno AUROC	0.71 (0.63–0.79)	0.70 (0.62–0.78)
Calibration, 5 years		
O/E probability of death within 5 years of diagnosis	1.15 (0.92–1.44)	1.19 (0.95–1.49)
Calibration slope at fixed 5‐year timepoint	0.70 (0.47–0.94)	0.70 (0.45–0.94)
ICI	0.052	0.057
E50	0.063	0.070
E90	0.085	0.089
5‐year model performance		
Brier score	0.220	0.221
IPA	9.3%	9.4%
Sensitivity	0.78 (0.69–0.88)	0.78 (0.69–0.88)
Specificity	0.57 (0.49–0.66)	0.57 (0.49–0.65)
PPV	0.50 (0.41–0.59)	0.50 (0.41–0.59)
NPV	0.83 (0.76–0.91)	0.83 (0.76–0.91)

Abbreviations: E50: median of the absolute difference between observed and predicted probabilities; E90: 90th percentile of the absolute difference between observed and predicted probabilities; ICI: Integrated Calibration Index; IPA: Index of Prediction Accuracy; O/E: Observed‐to‐expected.

* *p* < 0.001.

**FIGURE 2:**
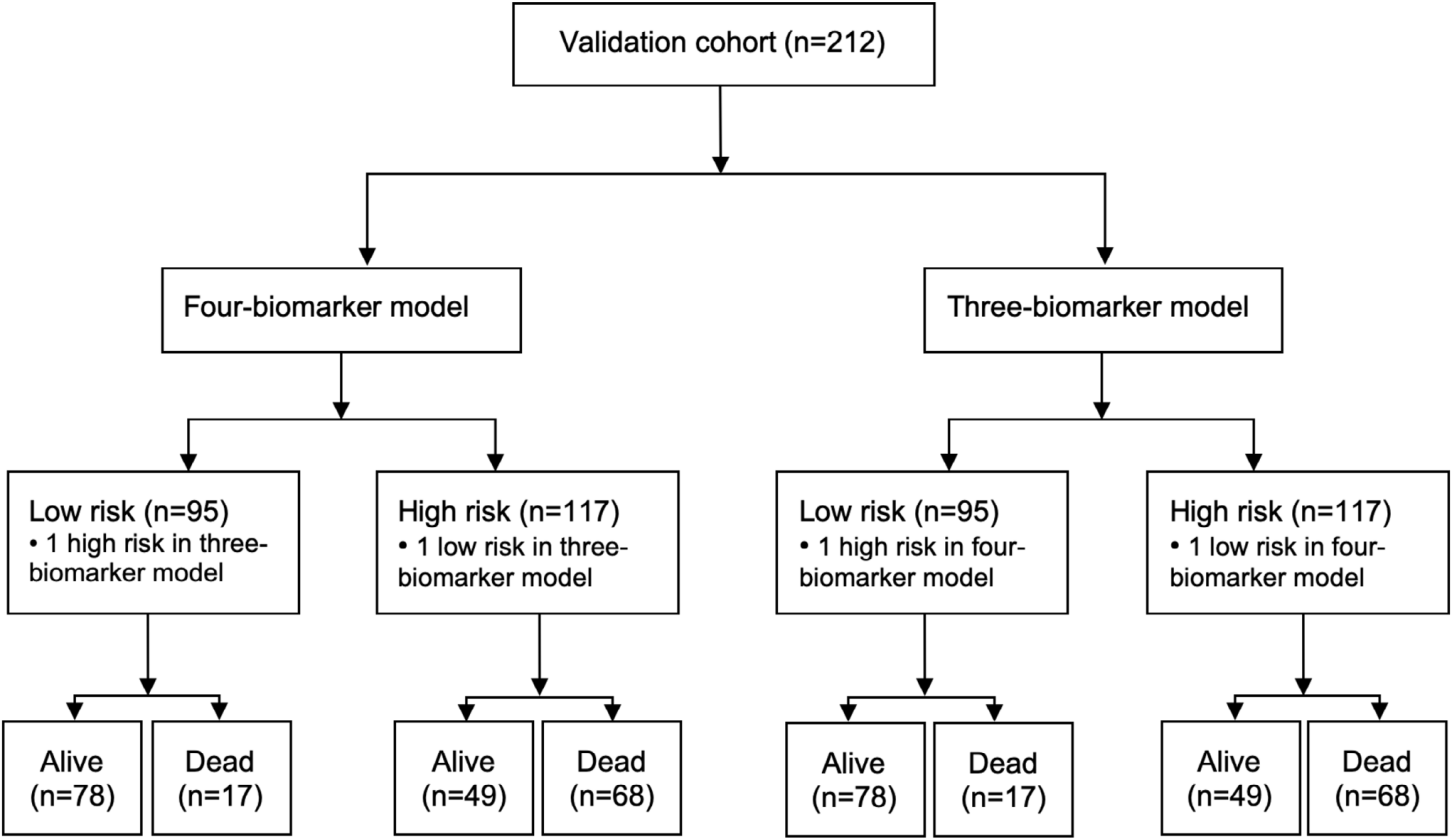
Risk group classifications and outcomes in the validation cohort by the four‐ and three‐biomarker models.

Both models assigned 210 (99.1%) participants to the same risk group, classifying 117 (55.2%) as high‐risk (Figure 2). One participant was classified as high‐risk by the three‐biomarker model but low‐risk by the four‐biomarker model; another participant was classified as low‐risk by the three‐biomarker model but high‐risk by the four‐biomarker model; both survived. Among 85 deaths (40.5%), both models identified 68 as high‐risk, giving an overall (through the whole follow‐up period) sensitivity of 0.80 (95% CI: 0.72–0.89). The overall specificity, PPV, and NPV of the two models were the same: 0.61 (95% CI: 0.53–0.70), 0.58 (95% CI: 0.50–0.68), and 0.82 (95% CI: 0.74–0.90), respectively (Table 2).

### 3.3 Treatment Comparisons

The three‐biomarker model was predictive of improved survival with surgical versus non‐surgical treatment in the high‐risk group across training and validation cohorts. In the validation cohort, the HR for those receiving surgery compared to those without was 0.42 (95% CI: 0.25–0.70, *p* < 0.001) in the high‐risk group, but did not reach statistical significance in the low‐risk group (HR = 0.78; 95% CI: 0.35–1.72, *p* = 0.54) (Table 2). In the four‐biomarker model, the HR for surgery compared to no surgery in the high‐risk group was 0.46 (95% CI: 0.28–0.74, *p* < 0.001), and in the low‐risk group the HR was 0.82 (95% CI: 0.30–2.23, *p* = 0.70).

Figure 1 shows the Kaplan–Meier survival curves for those treated with surgery or CRT only within high‐ and low‐risk groups. In the high‐risk group, the estimated 3‐year survival probability was 66% for surgery vs. 44% for those without, and the estimated 5‐year survival probability was 57% for surgery vs. 37% for those without. In the low‐risk group, survival was similar between treatments (3‐year: 91% vs. 84%; 5‐year: 79% vs. 78% for surgery vs. those without, respectively). Estimated survival rates by treatment and risk group for the three‐biomarker model at additional timepoints are provided in Table S3. Demographic and tumor characteristics of the high‐ vs. low‐risk groups are presented in Table S4.

Adjusting for year of diagnosis to account for treatment improvements over time yielded similar results across risk groups. The HR for surgery vs. those without was 0.45 (95% CI: 0.28–0.73, *p* = 0.001) in the high‐risk and 0.83 (95% CI: 0.31–2.26, *p* = 0.72) in the low‐risk group. No significant differences in risk factors (T‐category, N‐category, smoking status, and age at diagnosis) between treatment groups were found, except for T‐category in the high‐risk group (*p* < 0.05 after Benjamini‐Hochberg adjustment for multiple comparisons) (Table S5). After adjusting for treatment bias using propensity scores, HR was 0.32 (95% CI: 0.19–0.54, *p* < 0.001) in the high‐risk and 0.70 (95% CI: 0.41–3.97, *p* = 0.73) in the low‐risk group.

Sensitivity analysis restricted to patients treated with CRT (*n* = 84) showed that, in the high‐risk subgroup (*n* = 42), trimodality therapy remained associated with a lower risk of death (HR = 0.46, 95% CI: 0.17–1.26, *p* = 0.13 from the 4‐biomarker model; HR = 0.48, 95% CI: 0.16–1.41, *p* = 0.18 from the 3‐biomarker model). Median survival was 1.6 years for CRT but was not reached in the trimodality group. In the low‐risk subgroup (*n* = 42; 4 events), HR = 2.41 (95% CI: 0.28–20.54, *p* = 0.42) from the 4‐biomarker model and 2.53 (95% CI: 0.29–22.2, *p* = 0.40) from the 3‐biomarker model.

### 3.4 Model Comparisons

#### 3.4.1 Discrimination

The three‐biomarker model and the four‐biomarker model exhibited similar discrimination at 5 years in the validation cohort (Table 2). Uno's C‐index was 0.71 (95% CI: 0.65–0.76) for the three‐biomarker and 0.72 (95% CI: 0.66–0.78) for the four‐biomarker model. Harrell's C‐index yielded slightly higher estimates. Time‐dependent Uno's AUROC at 5 years was 0.70 (95% CI: 0.63–0.78) and 0.71 (95% CI: 0.66–0.79) for the three‐ and four‐biomarker models, respectively (Table 2). Discriminative measures at additional timepoints are presented in Table S5.

#### 3.4.2 Calibration

**FIGURE 3:**
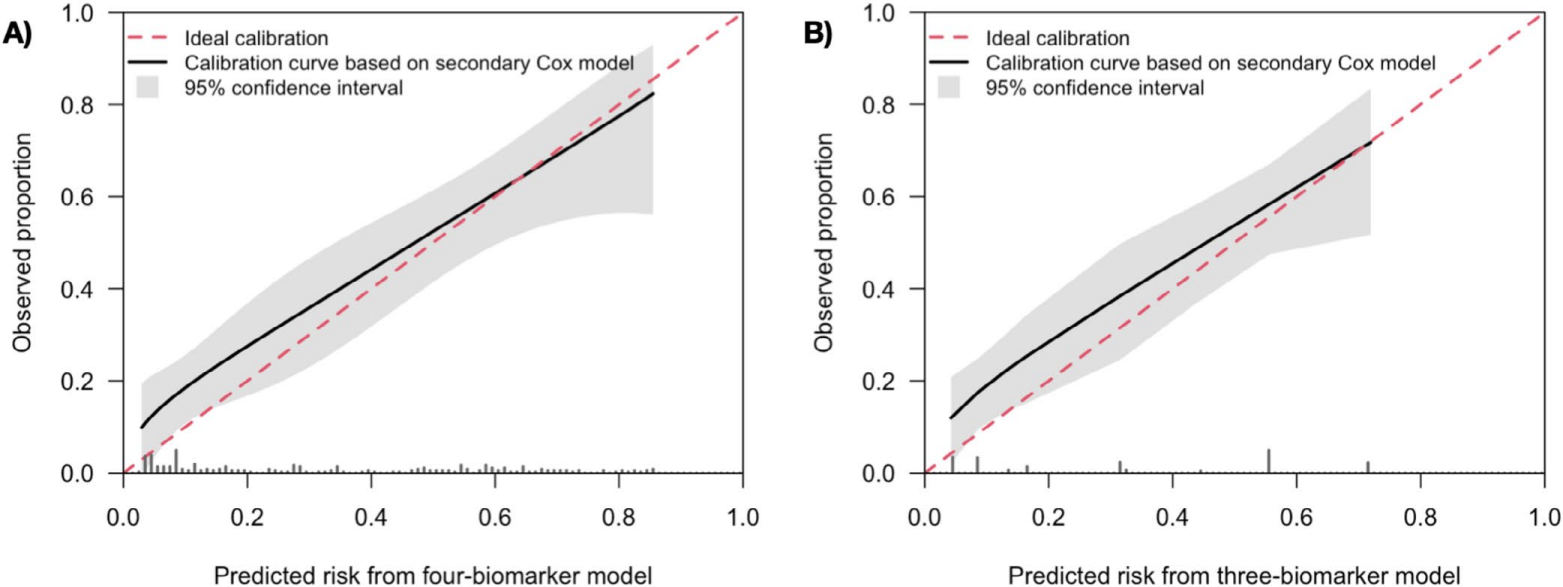
Calibration curves (black) with 95% CIs (gray shading) for the 5‐year risk in the validation cohort for the four‐ (A) and three‐biomarker (B) models. The ideal calibration line (red) goes through the origin with a slope of 1. Rug plots to show the distribution of predicted risk scores are displayed at the bottom along the *x*‐axis. [Color figure can be viewed in the online issue, which is available at www.laryngoscope.com]

Calibration curves (Figure 3) plotted the observed vs. predicted 5‐year risk of death in the validation cohort. The three‐ and four‐biomarker models yielded a similar Integrated Calibration Index (ICI) (0.06 vs. 0.05, respectively), E50 (0.07 vs. 0.06), and E90 (0.09 for both) (Table 2). For mean calibration, the mean predicted 5‐year mortality for the three‐biomarker was 32% (95% CI: 29%–36%), while the observed probability as estimated by the Kaplan–Meier method was 39% (95% CI: 31%–45%), giving an O/E ratio of 1.19 (95% CI: 0.95–1.49) (Table 2). The mean predicted 5‐year mortality for the four‐biomarker model was 34% (95% CI: 30%–37%), giving an O/E ratio of 1.15 (95% CI: 0.92–1.44).

Weak calibration, assessing alignment between predicted and observed 5‐year mortality risks, was similar for both models. The calibration slopes were 0.70 (95% CI: 0.45–0.94) for the three‐biomarker and 0.70 (95% CI: 0.47–0.94) for the four‐biomarker model (Table 2). The three‐biomarker model predicted a slightly narrower range of risk values (0.04–0.72 vs. 0.03–0.85 predicted by the four‐biomarker model).

#### 3.4.3 Overall Performance

The Brier score at 5 years was 0.22 for both the three‐ and four‐biomarker models (Table 2). The scaled Brier score, or IPA, was 9.4% for the three‐biomarker and 9.3% for the four‐biomarker model (Table 2). The likelihood ratio test yielded *p* = 0.087 and *p* = 0.066 in the training and validation sets, respectively, indicating that including survivin did not lead to statistically significant improvement in fit at the 0.05 level. 5‐year sensitivity, PPV, and NPV were the same in both models: 0.78 (95% CI: 0.69–0.88), 0.50 (95% CI: 0.41–0.59), and 0.83 (95% CI: 0.76–0.91), respectively (Table 2). The 5‐year specificity was nearly identical, with 0.57 (95% CI: 0.49–0.65) in the three‐biomarker model and 0.57 (95% CI: 0.49–0.66) in the four‐biomarker model (Table 2).

### 3.5 Clinical Utility

DCA showed that both models provided greater net benefit than the treat‐all strategy at a threshold probability (p_t_), the predicted probability of death at 5 years at which a clinician would recommend treatment escalation, greater than 0.22. The three‐biomarker model showed higher net benefit than no treatment at *p*_t_ ≤ 0.71, while the four‐biomarker model remained superior to no treatment up to *p*_t_ ≤ 0.85.

**FIGURE 4:**
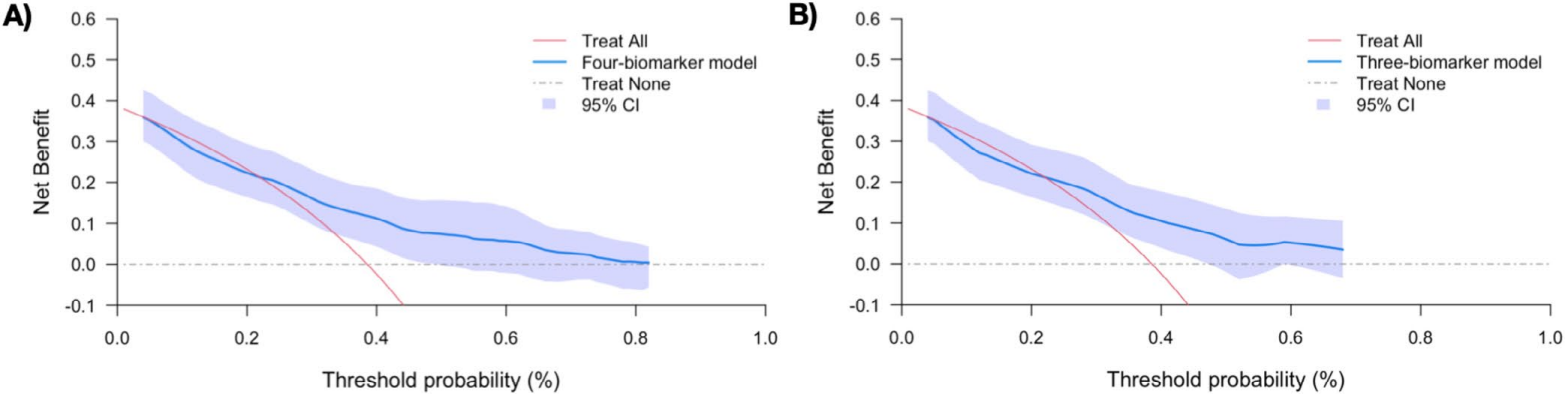
Decision curves with 95% CIs for the four‐biomarker (A) and three‐biomarker (B) models. [Color figure can be viewed in the online issue, which is available at www.laryngoscope.com]

The lower 95% CI for the three‐biomarker model exceeded treat‐all and no‐treatment strategies for 0.34 ≤ *p*_t_ ≤ 0.47, compared to 0.34 ≤ *p*_t_ ≤ 0.49 for the four‐biomarker model. Treat‐all was within 95% CIs at lower threshold probabilities (*p*_t_ < 0.34) for both models (Figure 4).

## 4 Discussion

Among prognostic models developed for OPSCC, PREDICTR‐OPC is one of very few that demonstrated predictive ability for treatment selection [9]. While HPV status and p16 expression provide important prognostic information, our prior analysis showed that clinical models incorporating these variables did not demonstrate comparable predictive ability to the PREDICTR‐OPC, suggesting that integrated biomarker‐based stratification offers additional guidance [9]. In this paper, we report that the updated three‐biomarker model (p16, HR‐HPV, TILs) displayed predictive and prognostic performance highly comparable to the original four‐biomarker classifier. Surgery was associated with significantly improved survival in the high‐risk group in both models. Model fit did not differ significantly, and sensitivity, specificity, PPV, and NPV were the same or nearly identical. Discrimination, calibration, and clinical utility were also similar.

The current study aimed to re‐evaluate the contribution of the biomarker survivin, given its relatively small effect size and practical limitations at implementation. Testing for p16 and HPV is already routine [29], and TILs are readily assessed using H&E staining [30]. In contrast, survivin was the only biomarker in the four‐biomarker model for which scoring could not be automated and showed moderate interobserver variability, restricting reproducibility and scalability. Removing survivin could therefore simplify the model and lower barriers to implementation, provided performance is maintained.

These results suggest that survivin has limited predictive ability. Its multiple splice variants, one of which—survivin‐2B—lacks antiapoptotic activity, may antagonize antiapoptotic isoforms and initiate mitochondrial apoptosis, and is overexpressed in oral cancers [31, 32, 33, 34, 35]. Standard IHC does not distinguish between isoforms, which may contribute to survivin's diminished prognostic value in our study.

There are some limitations of our study to address. As the models were developed using retrospective, non‐randomized data, they are subject to inherent biases, particularly selection bias and confounding. To mitigate these limitations, Mehanna et al. followed Transparent Reporting of a multivariable prediction model for Individual Prognosis or Diagnosis guidelines, including use of a reliable endpoint (death) and blinded data collection and biomarker scoring to enhance internal validity [9, 36]. However, residual confounding cannot be entirely excluded.

To address heterogeneity within the surgical cohort, we performed a sensitivity analysis restricted to patients treated with CRT. Within this subset, trimodality therapy remained associated with improved survival in the high‐risk group, although this did not reach statistical significance, likely reflecting reduced sample size. In the low‐risk group, event rates were low, limiting interpretability. Together, these findings support our primary results while underscoring the need for validation in larger cohorts.

A key strength of our study is that we have tested and compared the new and old models on the two original cohorts, with large sample sizes. Grade 3 external validation was performed on an independent cohort significantly differing in treatment and tumor characteristics; yet model performance remained consistent. We also applied extensive assessments of performance metrics and analyses.

Both models showed greater clinical utility than the treat‐all strategy for guiding the decision of surgical escalation when threshold probability (p_t_) for 5‐year mortality exceeded 22%, i.e., when the potential risks of surgery for up to four patients are considered an acceptable trade‐off to prevent undertreatment of one.

At lower mortality thresholds (*p*_t_ < 0.34), both models performed similarly to the treat‐all strategy, consistent with our finding that model‐guided treatment escalation offers limited advantages for low‐risk OPSCC patients. Although the four‐biomarker model showed higher net benefit over no‐treatment at a higher *p*_t_ (85%) compared to the three‐biomarker model (71%), reflecting a narrower range of predicted risk scores by the three‐biomarker model, this was not necessarily a true difference in utility. Furthermore, the 95% CI ranges for net benefit compared to default strategies were similar (four‐biomarker model: 34%–49%; three‐biomarker model: 34%–47%).

Currently, surgery is usually offered to low‐risk HPV + OPSCC cases that do not exhibit extranodal extension. However, the PREDICTR‐OPC model suggests that the addition of surgery is particularly effective in high‐risk OPSCC, thereby justifying the additional toxicity, whereas low‐risk patients may derive limited additional benefit. If validated, PREDICTR‐OPC could have important implications for current and future surgical practice.

## 5 Conclusions

In conclusion, we have found that removing survivin from the PREDICTR‐OPC classifier for OPSCC preserves prognostic and predictive ability, while improving scalability and reducing complexity. The three‐biomarker model has the potential to further reduce costs and facilitate implementation and maintains clinical utility to identify high‐risk OPSCC patients who may derive differential benefit from surgical escalation. Validation on a prospectively collected independent cohort is underway to confirm its clinical value.

## Funding

This work was supported by Cancer Research UK (C19677/A12617).

## Conflicts of Interest

Jill M. Brooks, Hisham Mehanna, and Catharine M. West report grants from Cancer Research UK during the conduct of the study. Terry M. Jones reports grants from GSK during the conduct of the study. Alice J. Sitch reports grants from MRC and NIHR Birmingham BRC during the conduct of the study. H. Mehanna reports grants from AstraZeneca, as well as other support from AstraZeneca, BMS, GSK, Johnson, Merck‐Serono, MSD, Nanobiotix, Seagen, Eisai Inc., Merck, Sanofi Pasteur, Warwickshire Head Neck Clinic, and Docspert outside the submitted work. Catharine M. West reports personal fees from Janssen outside the submitted work and being co‐founder of ManTRa Diagnostics, which was set up to commercialize hypoxia gene signatures; no financial remuneration received. The other authors declare no conflicts of interest.

## Supporting information


**Figure S1:** Kaplan–Meier curves for the high‐ and low‐risk groups from the four‐biomarker (A) and three‐biomarker models (B) in the training set.
**Figure S2:** Kaplan–Meier survival curves for the prognostic and predictive assessment of risk groups in the training cohort predicted by the four‐biomarker (A) and three‐biomarker models (B).
**Table S1:** Four‐ and three‐biomarker prognostic model for overall survival using training cohort data (*n* = 259, number of events = 71).
**Table S2:** Sensitivity, PPV, NPV, and C‐index for complete cases and imputed datasets.
**Table S3:** Overall model performance measures in the training cohort (*n* = 259).
**Table S4:** Estimated survival probability by the three‐biomarker model in the validation cohort by risk group and treatment strategy.
**Table S5:** Comparison of non‐surgical vs. surgical treatment groups in the three‐biomarker model by risk group, validation cohort.

## Data Availability

The data that support the findings of this study are available from the corresponding author upon reasonable request.
