# Supplementary material for: Refining the Multivariable Predictive‐Prognostic PREDICTR‐OPC Model for Survival in Surgical Escalation for Oropharyngeal Squamous Cell Carcinoma
Source: Laryngoscope. 2026 May 12;136(10):4290–300. doi: 10.1002/lary.70611 (PMC13569650; doi:10.1002/lary.70611)
Supplement: Supplementary file 1 [file LARY-136-4290-s001.docx]

**Supplementary Materials**

**Study Design and Data**

This study aims to refine the PREDICTR-OPC model by removing survivin and to compare the revised model’s performance to the original. The biomarker-only model was developed using a training cohort of 600 OPSCC patients treated with curative intent across ten UK and Polish centers from 1999 to 2012, with external validation on 385 patients from the HPV UK Prevalence study (2002-2011) (Mehanna et al., 2024; Schache et al., 2016). Tissue microarrays (TMAs) were constructed from formalin-fixed paraffin-embedded biopsies and stained for ten biomarkers that were independently scored by certified pathologists, including the four that were eventually selected for the model: p16, high-risk HPV (HR-HPV), survivin, and tumour-infiltrating lymphocytes (TILs) (Mehanna et al., 2024).

Each biomarker was independently scored by at least three pathologists who had previously undergone training and calibration. Survivin alone was treated as a continuous variable. HR-HPV was detected via in situ hybridization (ISH), resulting in binary classification. TILS were scored on whole sections stained with haematoxylin and eosin (H&E) and categorised as high (present in >80% of the tumour/stroma), moderate (present in 20-80% of the tumour/stroma) or low (present in <20% of the tumour/stroma). p16 and survivin were detected with immunohistochemical assays; scoring for immunohistochemistry was conducted by assessing the intensity and percentage of cells stained to produce an H-score of protein abundance ranging from 0 to 300. p16 was categorised as positive or negative based on a clinically validated cut-off translating to an H-score ≥ 140 (Ang et al., 2010). TILs were categorised as high, moderate, or low based on presence in >80%, 20-80%, or <20% of the tumour/stroma, respectively. In the training and validation datasets, H-scores were divided by 30 and standardised using Z-score transformation. Full details of the centres, staining protocols and biomarker scoring were previously detailed (Mehanna et al., 2024).

**Statistical Analysis**

Descriptive statistics included the mean and standard deviation for continuous variables and proportions for categorical variables. Model performance was assessed using discrimination, calibration, and clinical utility metrics, following STRATOS (STRengthening Analytical Thinking for Observational Studies) initiative recommendations (McLernon et al., 2023). Multiple imputations were performed based on all combinations of pmmtype (1 or 2) and boot.method ("simple" or "approximate bayesian") using aregImpute() from *Hmisc* (Harrell & Dupont, 2025). The revised model was retrained on these imputed datasets, and sensitivity, specificity, positive predictive value (PPV), negative predictive value (NPV), and C-index were compared to those produced by the complete-case dataset to evaluate the impact of missing data (results given in Supplementary Table S2). All analyses were performed in R (version 4.4.3).

**Model Training and Evaluation**

The previous four-biomarker PREDICTR-OPC model was revised by excluding survivin and fitting a Cox proportional hazards model with the remaining predictors (p16, HR-HPV ISH, and TILs) on the training cohort. Model coefficients are provided in Supplementary Table S1. The continuous H-score for survivin was divided by 30 and z-transformed (mean = 0, SD = 1), and p16 and TILs retained the cutoffs used in the original model. Participants were classified as high-risk if their risk score was greater than or equal to the training cohort median. The proportional hazards assumption for each covariate was assessed using Schoenfeld residuals, with the cox.zph function from the R package *survival* to plot residuals against log-transformed event time (Therneau et al., 2024).

After training, models were validated on the independent external validation cohort (n = 385).

**Risk Stratification and Treatment Strategy**

In the validation cohort, the multivariable Cox proportional hazards models assessed the association between risk group and OS, adjusting for age, year of diagnosis, T-category, N-category, smoking status, surgery, radiotherapy, and chemotherapy, producing estimated hazard ratios (HRs) and *P*-values. HRs were reported with the low-risk group as the baseline.

To compare OS for surgical vs. non-surgical treatment within risk groups, propensity score matching (PSM) was performed in the validation cohort to balance T-category, N-category, smoking status and age at diagnosis between treatment groups to adjust for treatment selection bias. PSM was conducted using *MatchIt* in R (method = "full", distance = "glm"), and the resulting weights were incorporated into a weighted Cox model to estimate marginal HRs for surgery vs. CRT (Ho et al., 2025). OS was compared by risk group and treatment for the three-biomarker model by generating Kaplan-Meier curves and comparing survival for the entire follow-up duration with the log-rank test (Supplementary Table S4).

**Model Comparison Metrics**

1. **Discrimination**

Discrimination is the ability of a model to distinguish between high- and low-risk patients. Time range discrimination, which evaluates how well a model differentiates between individuals experiencing an event within a specific time window, was assessed with Uno’s Concordance Index (C-index). This is the proportion of all possible pairs of patients for which the model correctly predicts the order of events occurring, and so higher values indicate better risk stratification. Uno’s C-index was used as it accounts for censored observations, unlike Harrell’s C-index, which becomes increasingly overconfident with more censoring (Blanche et al., 2019; Uno et al., 2011). Both were found at 3-, 5- and 10- year follow-up, with R packages *survival* and *concordance*, and compared with that of the original model (Supplementary Table S5).

Uno’s C-index assesses the model’s ability to rank survival times correctly up to a timepoint, capturing temporal ordering, while time-dependent discrimination evaluates accuracy in classifying event status at those exact timepoints. The time-dependent discrimination was assessed using the area under the receiver operating characteristic curve (AUROC) at 3-, 5- and 10-year timepoints (Supplementary Table S5). The receiver operating characteristic (ROC) curve plots the true positive rate (sensitivity) against the false positive rate (1-specificity). A value of 0.5 indicates no discrimination (random chance), while a value closer to 1.0 reflects perfect risk stratification (Park et al., 2021). Uno’s inverse probability of censoring weighting approach was used with the package *timeROC* (Blanche, 2019).

2. **Calibration**

Calibration is a crucial component of external validation that measures how closely predicted survival probabilities match observed survival rates. To address added complications due to censoring, we assessed calibration using three methods: mean, weak and moderate calibration. Calibration plots were created for visual assessment using the package *rms* (Harrell, 2025).

Mean calibration measures systematic underprediction or overprediction by taking the ratio of the observed probability of the event (death within 5 years) to the mean predicted probability. An observed-to-expected (O/E) ratio close to 1 with a narrow confidence interval indicates adequate mean calibration. The Kaplan-Meier estimate of 5-year survival in the external validation dataset was calculated with *survfit* and *summary* from the package *survival* and mean predicted probability at 5 years with the *predictRisk* from package *riskRegression* (Gerds et al., 2025). The O/E ratios were computed with 95% CIs.

Weak calibration extends mean calibration by evaluating whether observed outcomes align with predicted probabilities across different risks. This was assessed by estimating the calibration slope of a regression model in which the risk score was used as the only covariate in the validation dataset, with administrative censoring applied at 5 years. A slope of 1 indicates perfect calibration, while a slope below 1 suggests that low predicted risks are underestimated and high predicted risks are overestimated, and a slope above 1 indicates the reverse. For our models, a secondary Cox regression was fitted to determine the calibration slope with a 95% confidence interval using predict.coxph() and coxph() from package *survival*, then slopes were compared.

Moderate calibration assesses whether the observed event rate matches the predicted risk within subgroups of patients with similar predicted risk levels. Smooth calibration curves were evaluated by fitting a secondary Cox model, which regresses the observed survival outcome in the validation dataset on the 5-year risk score predicted by the development model. The comparison was supplemented by calculating both models’ Integrated Calibration Index (ICI), defined as mean absolute difference between the observed and predicted probabilities, and the E50 and E90, defined as the median and 90th percentile absolute difference between observed and predicted probabilities (Austin & Steyerberg, 2019). predictRisk() from *riskRegression* and coxph() from *survival* were used to predict 5-year risk and to apply the secondary Cox model, respectively.

3. **Model Fit and Accuracy**

Sensitivity, specificity, PPV, and NPV were calculated in the validation cohort after excluding patients censored before 5 years, comparing with those of the original model. The Likelihood Ratio (LR) test, which compares model fits by assessing whether the difference in log-likelihoods between them is statistically significant, was conducted. A significant LR test result (*P*<0.05) would indicate that survivin provides additional predictive value, while a non-significant result would suggest that its removal does not meaningfully impact model performance (Lee et al., 1983; Lewis et al., 2011).

Overall predictive accuracy was compared using the Brier score, which quantifies the mean squared error between predicted and actual survival outcomes, on training and validation cohorts; internal validation with bootstrapping were performed to adjust for optimism in the training cohort (Brier, 1950; Graf et al., 1999). The Brier score ranges from 0 to 0.25, where lower values indicate better predictive accuracy. For a more interpretable metric, we also computed the Index of Prediction Accuracy (IPA) or scaled Brier score, which expresses improvement over a null model assuming constant risk for all patients (Kattan & Gerds, 2018). The function Brier() was used from the package *SurvMetrics* (Zhou et al., 2023).

**Decision Curve Analysis**

To compare the clinical utility of the models, we performed Decision Curve Analysis (DCA) to evaluate their net benefit across a range of threshold probabilities (p_t_). DCA quantifies the trade-off between true positive and false positive classifications by incorporating the relative harms and benefits of intervention (Kerr et al., 2016; Vickers & Elkin, 2006). Unlike performance metrics for model discrimination and calibration, DCA calculates the net benefit of a model to determine whether its application improves clinical decision-making (Van Calster et al., 2018; Vickers et al., 2008). This can be compared to extreme strategies, i.e. performing surgery on all or no patients. The threshold probability (p_t_ ) is defined as the probability of death at which a clinician would consider recommending an intervention based on predicted risk. The net benefit at each threshold was calculated using the formula:

where TP and FP represent the number of true and false positive (high-risk) classifications, respectively, and N is the size of the validation cohort (Vickers & Elkin, 2006). The models were compared by plotting their net benefit across a clinically relevant range of threshold probabilities with the function stdca() from the *ggscidca* package, with higher net benefit indicating superior decision-making utility (Liu, 2024). The 95% CIs for net benefit predicted by both models were also calculated with bootstrapping using the package *boot*, and the threshold probabilities where these did not overlap with the default strategy curves were identified (Canty et al., 2024).

**Risk factor comparisons between low- and high-risk groups**

Comparison of risk factors T-category, N-category, smoking status, and age at diagnosis showed no significant differences between treatment groups across high- and low-risk participants, except for T category in the high-risk group (*P*<0.05 after adjustment for multiple comparisons using the Benjamini-Hochberg method to control the false discovery rate (Supplementary Table S3). High T-category (T3 or T4) was associated with receiving surgery.

Subgroup analysis identified T-category as a potential effect modifier: significant benefit with surgery was observed in T1-T2 disease, with HR of 0.30 (95% CI: 0.14-0.63, *P*=0.002), but not in T3-T4, with HR of 0.71 (95% CI: 0.37-1.38, *P*=0.31). Further adjustment for treatment bias using propensity scores based on the risk factors yielded HRs of 0.32 (95%CI: 0.19-0.54, P<0.001) in the high-risk and 0.70 (95% CI: 0.41-3.97, P=0.73) in the low-risk group. This is consistent with Mehanna et al.’s findings and may reflect higher risk of distant metastases in more advanced tumours, incurable by local surgical resection (Mehanna et al., 2024).

**References**

Ang, K. K., Harris, J., Wheeler, R., Weber, R., Rosenthal, D. I., Nguyen-Tân, P. F., Westra, W. H., Chung, C. H., Jordan, R. C., Lu, C., Kim, H., Axelrod, R., Silverman, C. C., Redmond, K. P., & Gillison, M. L. (2010). Human Papillomavirus and Survival of Patients with Oropharyngeal Cancer. *The New England Journal of Medicine*, *363*(1), 24–35. <https://doi.org/10.1056/NEJMoa0912217>

Austin, P. C., & Steyerberg, E. W. (2019). The Integrated Calibration Index (ICI) and related metrics for quantifying the calibration of logistic regression models. *Statistics in Medicine*, *38*(21), 4051–4065. <https://doi.org/10.1002/sim.8281>

Blanche, P. (2019). *timeROC: Time-Dependent ROC Curve and AUC for Censored Survival Data* (Version 0.4) [Computer software]. <https://cran.r-project.org/web/packages/timeROC/index.html>

Blanche, P., Kattan, M. W., & Gerds, T. A. (2019). The c-index is not proper for the evaluation of $t$-year predicted risks. *Biostatistics (Oxford, England)*, *20*(2), 347–357. <https://doi.org/10.1093/biostatistics/kxy006>

Brier, G. W. (1950). VERIFICATION OF FORECASTS EXPRESSED IN TERMS OF PROBABILITY. *Monthly Weather Review*, *78*(1), 1–3. [https://doi.org/10.1175/1520-0493(1950)078<0001:VOFEIT>2.0.CO;2](https://doi.org/10.1175/1520-0493(1950)078%3c0001:VOFEIT%3e2.0.CO;2)

Canty, A., Ripley, B., & Brazzale, A. R. (2024). *boot: Bootstrap Functions (Originally by Angelo Canty for S)* (Version 1.3-31) [Computer software]. <https://cran.r-project.org/web/packages/boot/index.html>

Gerds, T. A., Ohlendorff, J. S., Blanche, P., Mortensen, R., Wright, M., Tollenaar, N., Muschelli, J., Mogensen, U. B., & Ozenne, B. (2025). *riskRegression: Risk Regression Models and Prediction Scores for Survival Analysis with Competing Risks* (Version 2025.05.20) [Computer software]. <https://cran.r-project.org/web/packages/riskRegression/index.html>

Graf, E., Schmoor, C., Sauerbrei, W., & Schumacher, M. (1999). Assessment and comparison of prognostic classification schemes for survival data. *Statistics in Medicine*, *18*(17–18), 2529–2545. [https://doi.org/10.1002/(sici)1097-0258(19990915/30)18:17/18<2529::aid-sim274>3.0.co;2-5](https://doi.org/10.1002/(sici)1097-0258(19990915/30)18:17/18%3c2529::aid-sim274%3e3.0.co;2-5)

Harrell, F. E. (2025). *rms: Regression Modeling Strategies* (Version 8.0-0) [Computer software]. <https://cran.r-project.org/web/packages/rms/index.html>

Harrell, F. E., & Dupont, C. (2025). *Hmisc: Harrell Miscellaneous* (Version 5.2-3) [Computer software]. <https://cran.r-project.org/web/packages/Hmisc/index.html>

Ho, D., Imai, K., King, G., Stuart, E., Whitworth, A., & Greifer, N. (2025). *MatchIt: Nonparametric Preprocessing for Parametric Causal Inference* (Version 4.7.2) [Computer software]. <https://cran.r-project.org/web/packages/MatchIt/index.html>

Kattan, M. W., & Gerds, T. A. (2018). The index of prediction accuracy: An intuitive measure useful for evaluating risk prediction models. *Diagnostic and Prognostic Research*, *2*(1), 7. <https://doi.org/10.1186/s41512-018-0029-2>

Kerr, K. F., Brown, M. D., Zhu, K., & Janes, H. (2016). Assessing the Clinical Impact of Risk Prediction Models With Decision Curves: Guidance for Correct Interpretation and Appropriate Use. *Journal of Clinical Oncology*, *34*(21), 2534–2540. <https://doi.org/10.1200/JCO.2015.65.5654>

Lee, K. L., Harrell, F. E., Tolley, H. D., & Rosati, R. A. (1983). A comparison of test statistics for assessing the effects of concomitant variables in survival analysis. *Biometrics*, *39*(2), 341–350.

Lewis, F., Butler, A., & Gilbert, L. (2011). A unified approach to model selection using the likelihood ratio test. *Methods in Ecology and Evolution*, *2*(2), 155–162. <https://doi.org/10.1111/j.2041-210X.2010.00063.x>

Liu, Q. (2024). *ggscidca: Plotting Decision Curve Analysis with Coloured Bars* (Version 0.2.3) [Computer software]. <https://cran.r-project.org/web/packages/ggscidca/index.html>

McLernon, D. J., Giardiello, D., Van Calster, B., Wynants, L., van Geloven, N., van Smeden, M., Therneau, T., Steyerberg, E. W., & topic groups 6 and 8 of the STRATOS Initiative. (2023). Assessing Performance and Clinical Usefulness in Prediction Models With Survival Outcomes: Practical Guidance for Cox Proportional Hazards Models. *Annals of Internal Medicine*, *176*(1), 105–114. <https://doi.org/10.7326/M22-0844>

Mehanna, H., Rapozo, D., von Zeidler, S. V., Harrington, K. J., Winter, S. C., Hartley, A., Nankivell, P., Schache, A. G., Sloan, P., Odell, E. W., Thavaraj, S., Hunter, K. D., Shah, K. A., Thomas, G. J., Long, A., Amel-Kashipaz, R., Brown, R. M., Conn, B., Hall, G. L., … Robinson, M. (2024). Developing and Validating a Multivariable Prognostic-Predictive Classifier for Treatment Escalation of Oropharyngeal Squamous Cell Carcinoma: The PREDICTR-OPC Study. *Clinical Cancer Research*, *30*(2), 356–367. <https://doi.org/10.1158/1078-0432.CCR-23-1013>

Park, S. Y., Park, J. E., Kim, H., & Park, S. H. (2021). Review of Statistical Methods for Evaluating the Performance of Survival or Other Time-to-Event Prediction Models (from Conventional to Deep Learning Approaches). *Korean Journal of Radiology*, *22*(10), 1697–1707. <https://doi.org/10.3348/kjr.2021.0223>

Schache, A. G., Powell, N. G., Cuschieri, K. S., Robinson, M., Leary, S., Mehanna, H., Rapozo, D., Long, A., Cubie, H., Junor, E., Monaghan, H., Harrington, K. J., Nutting, C. M., Schick, U., Lau, A. S., Upile, N., Sheard, J., Brougham, K., West, C. M. L., … Jones, T. M. (2016). HPV-Related Oropharynx Cancer in the United Kingdom: An Evolution in the Understanding of Disease Etiology. *Cancer Research*, *76*(22), 6598–6606. <https://doi.org/10.1158/0008-5472.CAN-16-0633>

Therneau, T. M., until 2009), T. L. (original S.->R port and R. maintainer, Elizabeth, A., & Cynthia, C. (2024). *survival: Survival Analysis* (Version 3.8-3) [Computer software]. <https://cran.r-project.org/web/packages/survival/index.html>

Uno, H., Cai, T., Pencina, M. J., D’Agostino, R. B., & Wei, L. J. (2011). On the C-statistics for Evaluating Overall Adequacy of Risk Prediction Procedures with Censored Survival Data. *Statistics in Medicine*, *30*(10), 1105–1117. <https://doi.org/10.1002/sim.4154>

Van Calster, B., Wynants, L., Verbeek, J. F. M., Verbakel, J. Y., Christodoulou, E., Vickers, A. J., Roobol, M. J., & Steyerberg, E. W. (2018). Reporting and Interpreting Decision Curve Analysis: A Guide for Investigators. *European Urology*, *74*(6), 796–804. <https://doi.org/10.1016/j.eururo.2018.08.038>

Vickers, A. J., Cronin, A. M., Elkin, E. B., & Gonen, M. (2008). Extensions to decision curve analysis, a novel method for evaluating diagnostic tests, prediction models and molecular markers. *BMC Medical Informatics and Decision Making*, *8*(1), 53. <https://doi.org/10.1186/1472-6947-8-53>

Vickers, A. J., & Elkin, E. B. (2006). Decision Curve Analysis: A Novel Method for Evaluating Prediction Models. *Medical Decision Making*, *26*(6), 565–574. <https://doi.org/10.1177/0272989X06295361>

Zhou, H., Wang*, H., Wang, S., & Zou, Y. (2023). SurvMetrics: An R package for Predictive Evaluation Metrics in Survival Analysis. *The R Journal*, *14*(4), 252–263.

**Supplementary Tables**

Supplementary Table S1. Four- and three-biomarker prognostic model for overall survival using training cohort data (n= 259, number of events = 71).

|  | coef | exp(coef) | se(coef) | z | *P*-value | Significance |
| --- | --- | --- | --- | --- | --- | --- |
| Four-biomarker model |  |  |  |  |  |  |
| Survivin | 0.2188 | 1.2445 | 0.1269 | 1.724 | 0.08480 | . |
| p16=1 | -0.8909 | 0.4103 | 0.3006 | -2.964 | 0.00304 | ** |
| HPVISHHR=1 | -1.3833 | 0.2508 | 0.4831 | -2.924 | 0.00346 | ** |
| TILS=2 | -0.5246 | 0.5918 | 0.2714 | -1.933 | 0.05328 | . |
| TILS=3 | -1.2317 | 0.2918 | 0.3966 | -3.106 | 0.00190 | ** |
| Three-biomarker model |  |  |  |  |  |  |
| p16=1 | -0.7844 | 0.4564 | 0.2947 | -2.662 | 0.00776 | ** |
| HPVISHHR=1 | -1.4089 | 0.2444 | 0.4722 | -2.984 | 0.00285 | ** |
| TILS=2 | -0.4448 | 0.6410 | 0.2678 | -1.661 | 0.09676 | . |
| TILS=3 | -1.1870 | 0.3051 | 0.3984 | -2.980 | 0.00289 | ** |

Significance codes: *** : *P*<0.001, ** : *P*< 0.01, * : *P*<0.05, . : *P*< 0.1.

coef: fitted coefficients of the model (negative value indicates improved survival). exp(coef): exponent of the coefficients, otherwise known as the hazard ratio (HR). se(coef) = standard error of coefficients. z = Wald statistics' z-score, coef/se(coef). TILS: tumour-infiltrating lymphocytes); TILS = 1: low (baseline), TILS = 2: moderate TILS, TILS=3: high TILS

Supplementary Table S2. Sensitivity, PPV, NPV, and C-index for complete cases and imputed datasets

| **Dataset** | **Sensitivity** | **PPV** | **NPV** | **C-index** |
| --- | --- | --- | --- | --- |
| Complete cases validation | 0.80 | 0.65 | 0.79 | 0.77 |
| Imputed dataset  Validation results  pmmtype=1  boot.method=“simple” | 0.81 | 0.62 | 0.80 | 0.77 |
| Imputed dataset  Validation results  pmmtype=1  boot.method= “approximate  bayesian” | 0.80 | 0.62 | 0.80 | 0.77 |
| Imputed dataset  Validation results  pmmtype=2  boot.method=“simple” | 0.69 | 0.67 | 0.76 | 0.77 |
| Imputed dataset  Validation results  pmmtype=2  boot.method=“approximate  bayesian” | 0.79 | 0.62 | 0.80 | 0.77 |

Supplementary Table S2. Overall model performance measures in the training cohort (n=259)

|  | Four-biomarker model | Three-biomarker model |
| --- | --- | --- |
| Sensitivity | 0.86 (0.78-0.93) | 0.86 (0.77-0.93) |
| Specificity | 0.64 (0.56-0.69) | 0.63 (0.56-0.69) |
| PPV | 0.47 (0.38-0.54) | 0.47 (0.38-0.54) |
| NPV | 0.92 (0.87-0.96) | 0.92 (0.87-0.96) |

Of 259 participants, 188 survived and 71 died.

Supplementary Table S3. Estimated survival probability by the three-biomarker model in the validation cohort by risk group and treatment strategy

| Time (y) | Low risk,  -surgery (n=35) | Low risk, +surgery (n=60) | High risk, -surgery (n=52) | High risk, +surgery (n=65) |
| --- | --- | --- | --- | --- |
| 1 | 0.939 (0.861-1.00) | 1.00 (1.00-1.00) | 0.673 (0.557-0.813) | 0.831 (0.744-0.927) |
| 2 | 0.905 (0.807-1.00) | 0.948 (0.893-1.00) | 0.507 (0.386-0.667) | 0.723 (0.622-0.840) |
| 3 | 0.835 (0.712-0.979) | 0.913 (0.843-0.989) | 0.439 (0.319-0.604) | 0.662 (0.556-0.787) |
| 4 | 0.835 (0.712-0.979) | 0.872 (0.787-0.966) | 0.366 (0.250-0.535) | 0.628 (0.521-0.758) |
| 5 | 0.779 (0.633-0.960) | 0.794 (0.685-0.920) | 0.366 (0.250-0.535) | 0.566 (0.454-0.707) |

Supplementary Table S4. Demographic and tumor characteristics by risk group.

| Variable | Low-risk N = 95*^1^* | High-risk N = 117*^1^* | ***P*-value***^2^* | **Adjusted *P*-value (BH)** |
| --- | --- | --- | --- | --- |
| **T** |  |  | 0.00327 | 6.538077e-03 |
| T1-T2 | 67 (71%) | 59 (50%) |  |  |
| T3-T4 | 28 (29%) | 58 (50%) |  |  |
| **N** |  |  | 0.01787 | 2.144067e-02 |
| N0-N2a | 45 (47%) | 75 (64%) |  |  |
| N2b-N3 | 50 (53%) | 42 (36%) |  |  |
| **Smoking status** |  |  | 8.52e-08 | 2.556247e-07 |
| Never | 37 (39%) | 15 (13%) |  |  |
| Previous | 36 (38%) | 33 (28%) |  |  |
| Current | 22 (23%) | 69 (59%) |  |  |
| **HR HPV** |  |  | < 2e-16 | 1.119666e-42 |
| 0 | 15 (16%) | 117 (100%) |  |  |
| 1 | 80 (84%) | 0 (0%) |  |  |
| **Gender** |  |  | 0.00650 | 9.749712e-03 |
| Female | 15 (16%) | 38 (32%) |  |  |
| Male | 80 (84%) | 79 (68%) |  |  |
| **Age** | 56 (10) | 59 (11) | 0.03469 | 3.468725e-02 |

^1^n (%); Mean (SD)
^2^Fisher’s exact test; Wilcoxon rank sum test

Supplementary Table S5. Comparison of non-surgical vs surgical treatment groups in the three-biomarker model by risk group, validation cohort

| **Variable** | **High-risk, no surgery**  N = 52*^1^* | **High-risk, Surgery**  N = 65*^1^* | **Adjusted p-value (BH)** | **Low-risk, no surgery**  N = 35*^1^* | **Low-risk, surgery**  N = 60*^1^* | **Adjusted p-value (BH)** |
| --- | --- | --- | --- | --- | --- | --- |
| **T** |  |  | 0.004 |  |  | 0.05 |
| 1 | 17 (33%) | 42 (65%) |  | 19 (54%) | 48 (80%) |  |
| 2 | 35 (67%) | 23 (35%) |  | 16 (46%) | 12 (20%) |  |
| **N** |  |  | 0.71 |  |  | 0.83 |
| 1 | 35 (67%) | 40 (62%) |  | 16 (46%) | 29 (48%) |  |
| 2 | 17 (33%) | 25 (38%) |  | 19 (54%) | 31 (52%) |  |
| **Smoking.status** |  |  | 0.054 |  |  | 0.83 |
| 0 | 4 (7.7%) | 11 (17%) |  | 12 (34%) | 25 (42%) |  |
| 1 | 10 (19%) | 23 (35%) |  | 14 (40%) | 22 (37%) |  |
| 2 | 38 (73%) | 31 (48%) |  | 9 (26%) | 13 (22%) |  |
| **HPVISHHR** |  |  | - |  |  | 0.83 |
| 0 | 52 (100%) | 65 (100%) |  | 6 (17%) | 9 (15%) |  |
| 1 | 0 (0%) | 0 (0%) |  | 29 (83%) | 51 (85%) |  |
| **Gender** |  |  | 0.71 |  |  | 0.83 |
| F | 19 (37%) | 19 (29%) |  | 7 (20%) | 8 (13%) |  |
| M | 33 (63%) | 46 (71%) |  | 28 (80%) | 52 (87%) |  |

*^1^* n (%)

**Supplementary Figures**

Supplementary Figure S1. Kaplan-Meier curves for the high- and low-risk groups from the four-biomarker (A) and three-biomarker models (B) in the training set.

Supplementary Figure S2. Kaplan-Meier survival curves for the prognostic and predictive assessment of risk groups in the training cohort predicted by the four-biomarker (A) and three-biomarker models (B).
